# Distinctive serum lipidomic profile of IVIG-resistant Kawasaki disease children before and after treatment

**DOI:** 10.1371/journal.pone.0283710

**Published:** 2023-03-29

**Authors:** Zhen Chen, Shuji Sai, Kiyoshi Nagumo, Yue Wu, Hitoshi Chiba, Shu-Ping Hui

**Affiliations:** 1 School of Pharmacy, Jiangsu University, Zhenjiang, Jiangsu, China; 2 Faculty of Health Sciences, Hokkaido University, Sapporo, Hokkaido, Japan; 3 Department of Pediatrics, Teine-Keijinkai Hospital, Sapporo, Hokkaido, Japan; 4 Institute for Genetic Medicine, Hokkaido University, Sapporo, Hokkaido, Japan; 5 Department of Nutrition, Sapporo University of Health Sciences, Sapporo, Hokkaido, Japan; Institut National de la Santeet de la Recherche Medicale (INSERM), FRANCE

## Abstract

Kawasaki Disease (KD) is an acute inflammatory disorder associated with systemic vasculitis. Intravenous immunoglobulin (IVIG) is an effective therapy for KD, yet, about 20% of cases show IVIG resistance with persistent inflammation. The lipid profile in IVIG-resistant KD patients and the relationship between lipid characteristics and IVIG resistance remain unknown. In this study, serum samples from twenty KD patients with different IVIG responses (sensitive, intermediate, or resistant) were collected both before and after treatment, and lipidomic analysis was performed using high-performance liquid chromatography-mass spectrometry. As a result, before treatment, six lipid species were found as the most variant features, in which all the top decreased lipids in the IVIG-resistant group were lysophosphatidylcholine (LPC) and lysophosphatidylethanolamine (LPE), suggesting the potential to be IVIG-resistant markers in pretreatment diagnosis. During treatment, lipidomic changes showed a weaker response in the IVIG-resistant group. After treatment, LPC and LPE species exhibited lower in the IVIG-resistant group and negative correlation with the inflammatory markers, indicating that the unique metabolism may occur among IVIG-responsiveness. These results might contribute to diagnosing IVIG-resistant patients more accurately for alternative therapy and to a better understanding of how lipid metabolism is associated with IVIG sensitiveness/resistance in KD.

## 1. Introduction

Kawasaki disease (KD) is an acute self-limited vasculitis that has become one of the leading causes of pediatric acquired heart disease in children [1–4]. Without early treatment, there is an approximately 25% chance of serious cardiovascular damage, including irreversible damage to the coronary arterial wall and even myocardial infarction [5, 6]. Intravenous immunoglobulin (IVIG) therapy can resolve inflammation in about 80% of KD cases [7]. However, 15%–25% of patients exhibit IVIG resistance, leading to persistent or recurrent fever [8–10], expressing a higher risk of developing coronary artery aneurysms [11]. The more extended the inflammation continues, the higher the risk of severe damage to the heart and blood vessels occurring with further complications. Therefore, early diagnosis and appropriate therapy are critical to reducing complications of KD, such as coronary artery aneurysms. Consequently, it is crucial to identify which patients show IVIG resistance and require additional therapy to reduce the risk of coronary artery injury [11].

There have been proposed some clinical risk-scoring systems for predicting the IVIG-resistant cases based on the clinical data before IVIG treatment, such as Gunma (Kobayashi), Kurume (Egami), and Osaka (Sano) scorings, which were mainly based on pathologic examination, proteins, and other clinical indexes, such as the increased neutrophil count, the decreased platelet count, hyponatremia, hepatic dysfunction, and elevated C-reactive protein (CRP) [11–13]. Our interest was to investigate lipid metabolism as another aspect of this issue because lipids serve as the structural components of biomembranes, store energy, deliver molecule signaling [14, 15], and participate in inflammatory response diseases [16, 17]. Despite that lipid homeostasis regulation has been well-known to play an essential role in various metabolic disorders [18], investigations on lipids are barely for KD [19, 20]. Recently, proteomics and metabolomics, have been applied to identify KD [21], but such approaches have yet to be reported on IVIG resistance in KD. A recent study discovered that oxidized phospholipids were involved in the pathogenesis of coronary arteritis in KD via activating the inflammatory signals [20]. However, the distinctive comparison of lipid characteristics in IVIG responsiveness has not been studied yet. Moreover, it remains unknown how different the IVIG-resistant patients exhibit lipidomic alterations during the treatment and whether they show differential lipid profiles after treatment.

Herein, we focused on lipid metabolism to provide new insights into IVIG responsiveness in KD patients. A comprehensive lipidomic analysis was conducted using liquid chromatography (LC) coupled with high-resolution mass spectrometry (HR-MS). The association with IVIG-sensitiveness/resistance to lipidomic profile was revealed to identify potential diagnostic markers for IVIG resistance and elucidate characterized lipid changes in IVIG-resistant KD patients during treatment.

## 2. Methods

### 2.1. Chemicals

The authentic internal standards (IS) for lipidomic analysis, namely, phosphatidylcholine (PC) 13:0/13:0, phosphatidylethanolamine (PE) 15:0/15:0, phosphatidylinositol (PI) 8:0/8:0, lysophosphatidylcholine (LPC) 15:0, lysophosphatidylethanolamine (LPE) 13:0, and lysophosphatidylinositol (LPI) 13:0, were purchased from Avanti Polar Lipids (Alabaster, AL), while triacylglycerol (TG) 11:0/11:0/11:0 and free fatty acid (FFA) 17:0 were obtained from Sigma-Aldrich (St. Louis, MO). Unless specified, other chemicals and reagents were of the highest grade available and purchased from Sigma-Aldrich.

### 2.2. IVIG-responsiveness of KD patients and serum sample collection

This study included twenty children diagnosed with KD and treated in the Department of Pediatrics, Teine-Keijinkai Hospital (Sapporo, Japan). The study was approved by the institutional review board of Teine-Keijinkai Hospital (Approval No.: 2015–009) and the Faculty of Health Sciences, Hokkaido University (Approval No.: 16–10), and was conducted according to the principles of the Declaration of Helsinki. Written informed parental consent was obtained from each patient.

All the patients were firstly treated with IVIG treatment (2 g/kg over 24 h) initiated on day 5 or 6 after the onset of high fever. IVIG sensitiveness/resistance was determined from the initial response to IVIG treatment according to the resolution of fever (< 38°C). Amongst them, ten were classified as IVIG-sensitive (Sen) patients, who showed complete resolution of high fever within 48 h after commencing IVIG treatment and remained afebrile. Five patients were classified IVIG-resistant (Res) due to persistent fever > 38°C following IVIG treatment and required additional treatments. And five patients were classified IVIG-intermediate (Int) due to persistent fever > 38°C over 48 h after IVIG treatment but did not require additional treatment as the fever went down spontaneously. Their corresponding clinical characteristics are listed in S1 Table.

The determination of white blood cell (WBC) and C-reactive protein (CRP), and the collection of peripheral blood samples, were conducted before (day 4 after the onset of high fever) and after IVIG treatment (day 8). Thereafter, the serum samples were separated by centrifugation within 30 min of collection, and thereafter stored at –80°C until analyzed.

### 2.3. Serum total lipid extraction

The extraction procedure was according to Bligh & Dyer [22] as described previously [23]. In Brief, 100 μL of plasma serum was extracted with 800 μL of ice-cold chloroform/methanol 1:1 (v/v, with IS and butylated hydroxytoluene) twice, followed by the dryness under vacuum. Then, the dried lipids were dissolved in 100 μL of methanol and centrifuged at 680 × g under 4°C for 15 min to remove any insoluble material prior to LC/MS injection. All the sample preparation was completed within 1 h to avoid lipid degradation and oxidation.

### 2.4. LC/MS-based lipidomics

The lipidomic analysis was carried out on a Shimadzu Prominence HPLC (Shimadzu Corp., Kyoto, Japan) coupled with an LTQ Orbitrap mass spectrometer (Thermo-Fisher Scientific Inc., San Jose, CA) operated in both electrospray ionization (ESI) positive and negative ionization modes, previous described [23, 24]. A Luna C18(2) column (2 mm × 150 mm, 3 μm, Phenomenex, Ltd, Torrance, CA) was equipped for chromatographic separation, with the column oven temperature holding at 40°C. The mobile phase consisted of 10 mM aqueous ammonium acetate (A), isopropanol (B), and methanol (C) at the flow rate of 200 μL/min, and the detailed gradient elution under positive and negative modes are shown in S2 Table.

For MS experiments, the spray voltage was set at 3.0 kV, and the sheath gas (nitrogen) and auxiliary gas (nitrogen) were 50 psi and 5 psi, respectively. The HR-MS^1^ data were acquired under Fourier transform (FT) mode for a full scan with the resolution power of 60,000, for which the scan ranges were set at *m/z* 150–1100 and *m/z* 220–1650 under positive mode and negative mode, respectively. While the MS^2^ fragmentation was conducted by collision-induced dissociation (CID) under Ion-trap (IT) mode, with the normalized collision energy set at 35% and the isolation width set at 2 Da. The data acquisition sequence was randomized to minimize the running time-induced variation.

The raw data were processed by the workstation Xcalibur 2.2 (Thermo-Fisher Scientific Inc.), with the accuracy of HR-MS^1^ within 5.0 ppm and MS^2^ within 0.5 Da. The identification was accorded to retention behavior in reversed-phase LC, HR-MS^1^ data, and MS^2^ fragments compared with LIPIDMAPS (www.lipidmaps.org). The annotated lipid species were indicated as follows: “lipid class + number of acyl carbon atoms + number of acyl double bonds” [25]. For semi-quantitation, the peak of the extracted ion chromatogram for each lipid species was integrated, and the amount of each lipid species was calculated by peak area normalization using the corresponding IS [26], as shown in the equation below.


$${Amount}_{Analyte}={Amount}_{IS}\times \frac{{Peak\;area}_{Analyte}}{{Peak\;area}_{IS}}$$

### 2.5. Statistical analysis

All the data were expressed as mean and standard deviation (SD). For group-wise comparisons, the Shapiro−Wilk test followed by Welch’s t-test, Mann−Whitney test, paired or unpaired Student’s t-test, and ANOVA were performed by using SPSS 24 (SPSS Inc., Chicago, IL) and GraphPad Prism 8 (La Jolla, CA), and *P*-value < 0.05 was regarded as existing of significant statistical difference. The diagnostic performance was evaluated based on the area under the receiver operating characteristic (ROC) curve (AUC) using JMP 14 (SAS Institute Inc., Cary, NC), and the optimal cut-off of distal baseline impedance was selected to maximize the sum of the sensitivity and specificity. The heatmap was generated using R 4.0 (www.r-project.org) with Euclidean clustering distance and Ward’s clustering method [25]. The correlation between variables was assessed by calculating Pearson’s correlation coefficient using SPSS 24.

## 3. Results

### 3.1. Identification and comparison of the lipid classes and molecular species

The present LC/MS and MS/MS analysis annotated a total of 119 lipid molecular species according to their LC retention behavior and ion peaks on HRMS (protonated ions [M + H]^+^, for PC and LPC, ammoniated ions [M + H]^+^ for TG, deprotonated ions [M − H]^−^ for FFA, PE, LPE, PI, and LPI). The fatty acyl composition was further identified based on the fragmentation of MS/MS (shown in S3 Table), and the semi-quantitative concentration of each lipid species was listed as a dataset in S4 Table.

**Fig 1:**
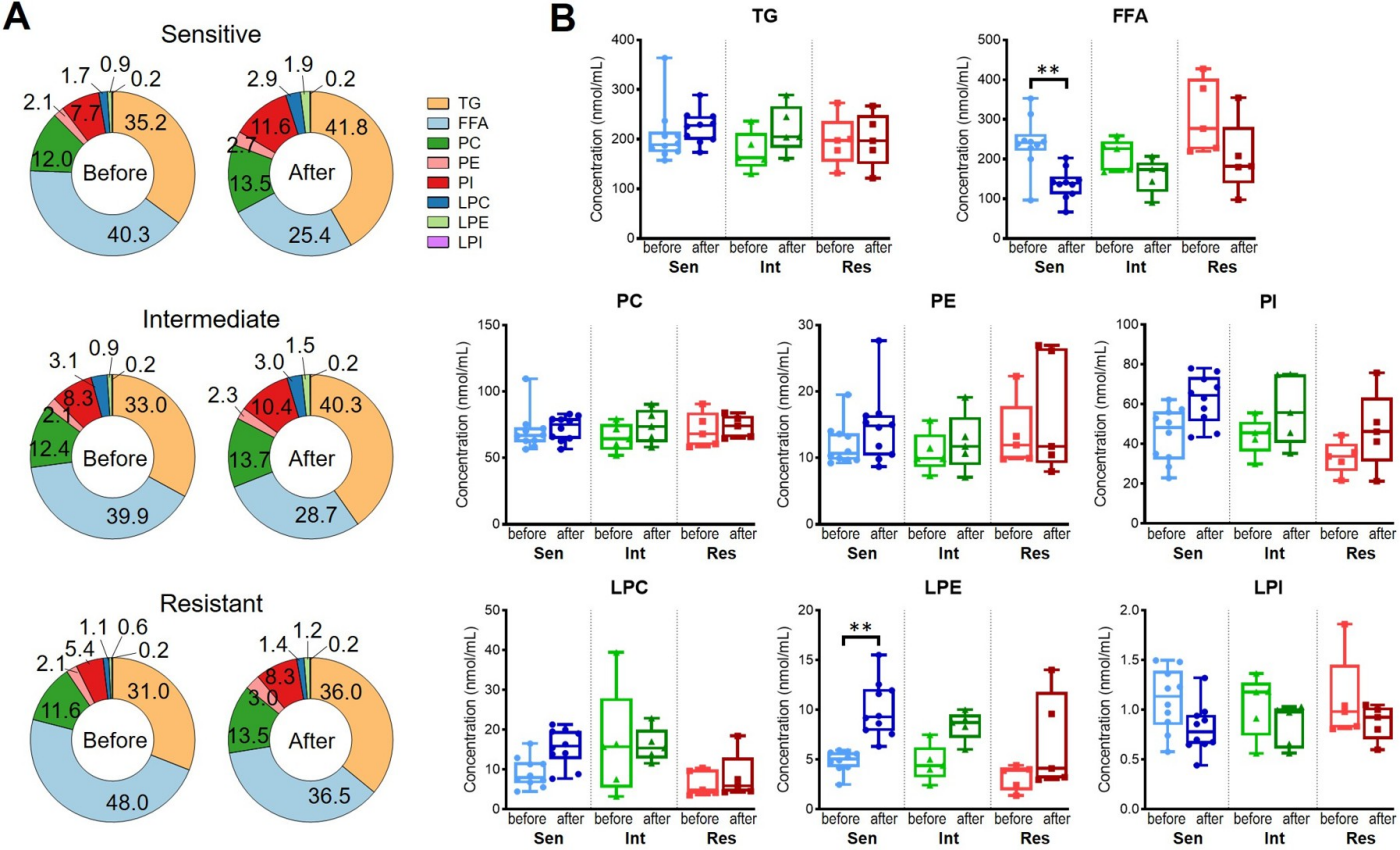
Percentage (A) and concentration (B) of every lipid class in the KD serum before and after treatment among IVIG-sensitive, -intermediate, and -resistant groups. ** *P* < 0.01.

The total amount of every lipid class and their composition are shown in Fig 1. TG and FFA were the predominant lipids, ranging from 31.0%–41.8% and 25.4%–48.0% of all the investigated lipids, respectively, followed by PC (11.6%–13.5%) and PI (5.4%–11.6%). While lysophospholipids, including LPC, LPE, and LPI, accounted for no more than 5% in all the groups (Fig 1A). For the content of every lipid class, no statistically significant differences were found among different IVIG groups. While in terms of the comparison between the before- and the after-treatment groups of the same IVIG responsiveness, the IVIG-sensitive groups showed a significant decrease in FFA (*P* = 0.009) and a significant increase in LPE (*P* = 0.002) (Fig 1B).

### 3.2. Characteristic lipid variations in IVIG-resistant patients before treatment

**Fig 2:**
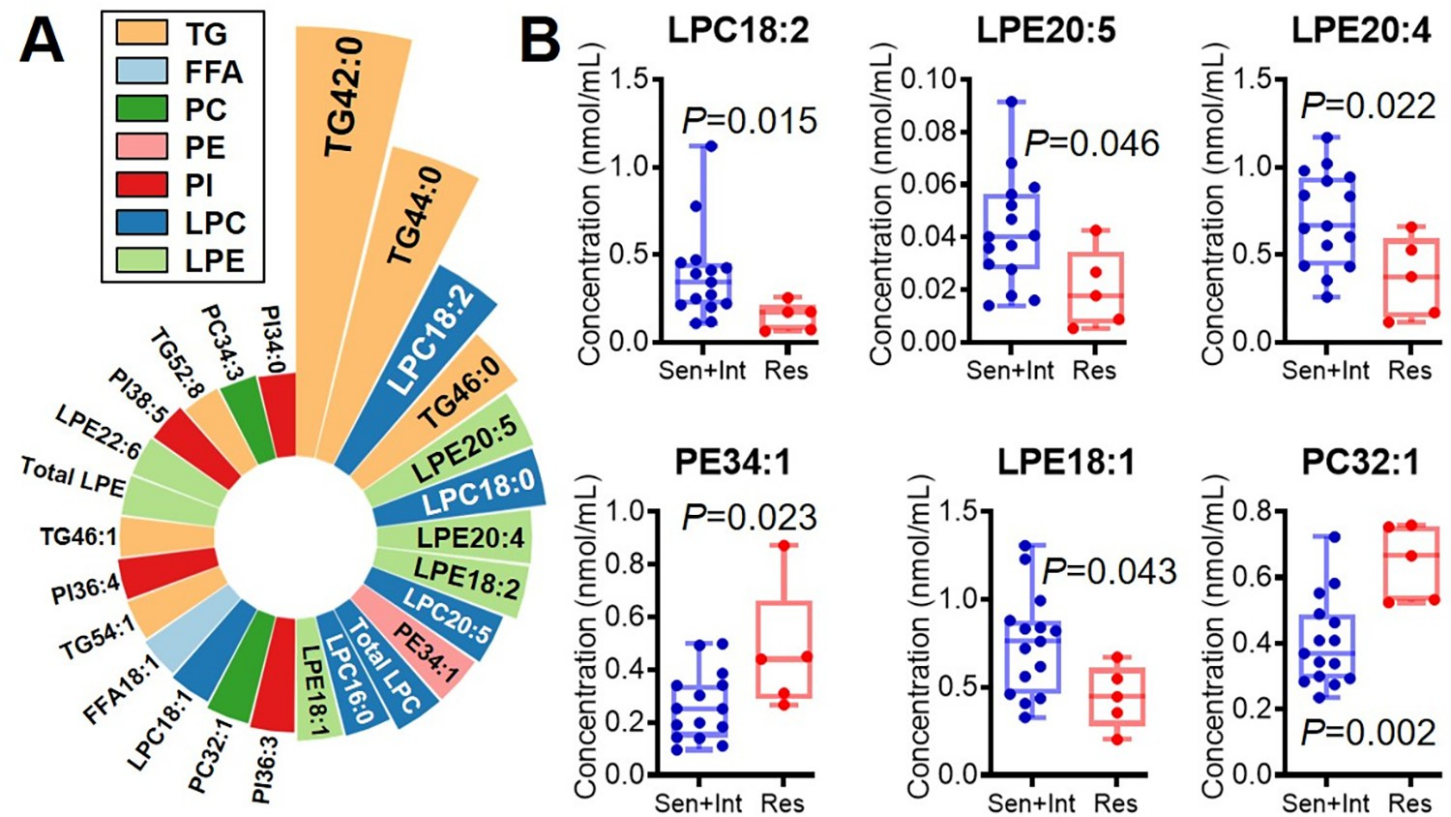
The Nightingale’s rose diagram of the top differentiated lipid species (A) and the comparison of the distinguished lipid species (B) between IVIG-sensitive + intermediate and IVIG-resistant groups before treatment.

For improving the KD pretreatment diagnosis and distinguishing the IVIG-resistant KD patients before treatment, all the before-treatment serum samples were divided into “Sen + Int” and “Res” groups. Then, the ratios of Sen + Int to Res for each lipid species were calculated (Fig 2A), in which the significantly differentiated lipid species were compared (Fig 2B). In the Res group, PE34:1 and PC32:1 were more enriched than Sen + Int group, accounting for 1.79 and 1.60 folds, respectively, while the lysophospholipids LPC18:2, LPE20:5, LPE20:4, and LPE18:1 were reduced by 40.4%–61.8%.

**Fig 3:**
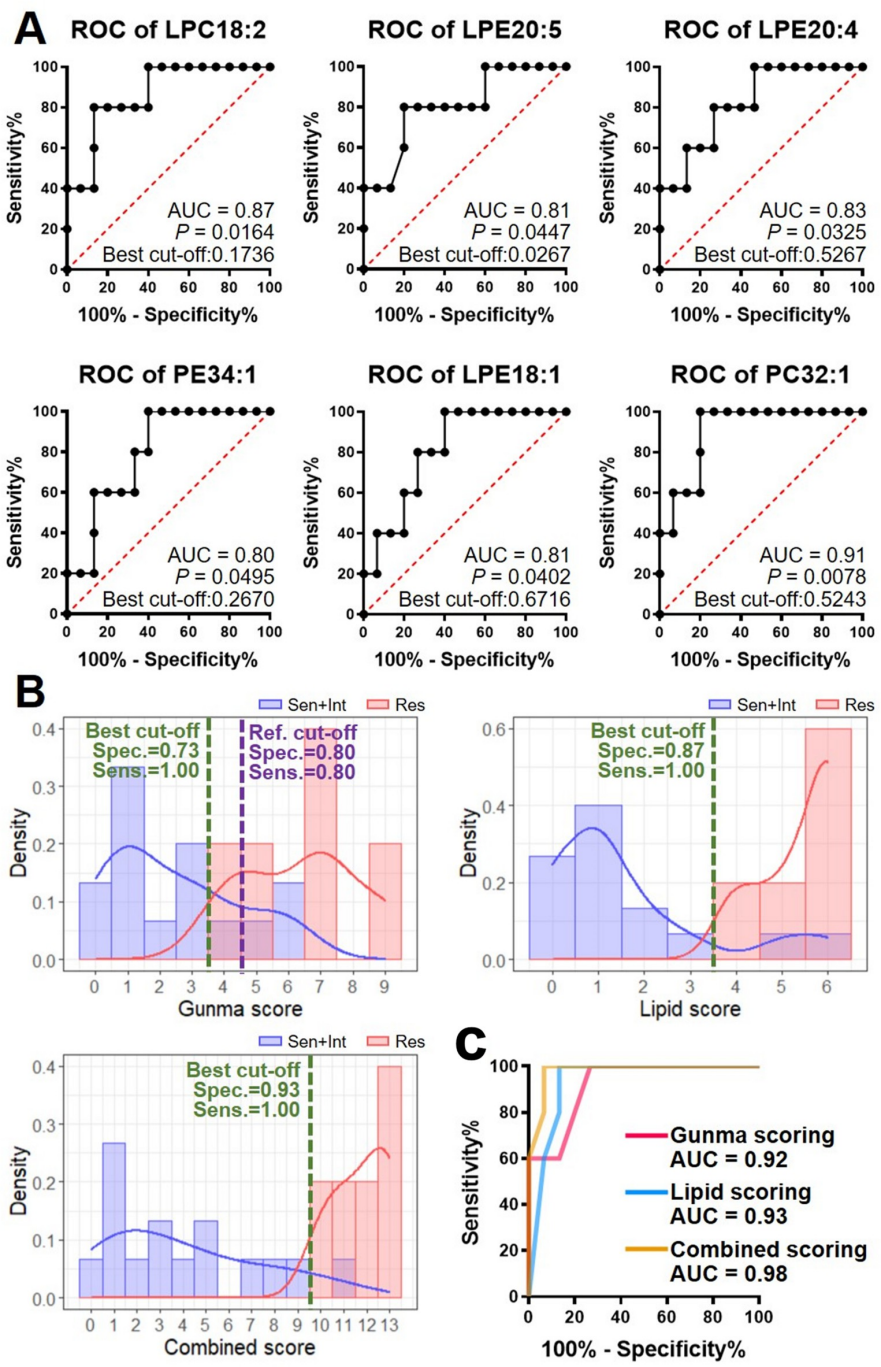
(A) ROC curves of these lipid species for diagnosing IVIG resistance, with AUC, *P*-value of the curve, and the best cut-off. (B) The histograms of IVIG-sensitive + intermediate (blue) and IVIG-resistant (red) patients based on Gunma scoring, lipid scoring, and combined scoring, respectively. The purple dashed line indicates the referred cut-off of Gunma score, and the green dashed lines indicate the best cut-off calculated for the current samples. (C) ROC curves of Gunma scoring, lipid scoring, and combined scoring for IVIG resistance diagnosis.

Moreover, the diagnostic power of each lipid species as the potential marker was evaluated. Their ROC curves (Fig 3A) showed that the six potential lipid markers exhibited promising capabilities, with AUC not less than 0.80 and *P*-value no more than 0.05 for all. The best cut-off, together with the corresponding specificity and sensitivity for each lipid species, was calculated, of which PC32:1 showed the most satisfied diagnostic capacity (best cut-off: 0.5243, with the specificity of 0.80 and sensitivity as 1.00). The results suggest that these lipids may predict IVIG responsiveness in KD.

Subsequently, by using these optimal cut-offs, we proposed a criterion (as the “lipid score”) based on these six lipid species (detailed criteria and scores are listed in S5 Table). Then, we compared the diagnostic capacities using Gunma scoring, lipid scoring, and a combination of both (Fig 3B and 3C). Gunma scoring showed decent capacity (0.80 for both specificity and sensitivity, 0.92 for AUC of ROC) at the reference standard (point ≥ 5) [11] and a comparable power (specificity, 0.73; sensitivity, 1.00) at the optimal cut-off (point ≥ 4, optimized for the current study). While lipid scoring also exhibited promising specificity (0.87) and sensitivity (1.00) under the threshold (best cut-off) of lipid score point ≥ 4 as IVIG resistance (with the AUC of 0.93). Furthermore, the combined scoring, i.e., the sum of Gunma score and lipid score, showed even boosted diagnostic efficiency (point ≥ 10 as IVIG-resistance threshold, 0.93 for specificity, 1.00 for sensitivity, and 0.98 for AUC).

### 3.3. Distinctive lipidomic changes among IVIG-response types during KD treatment

**Fig 4:**
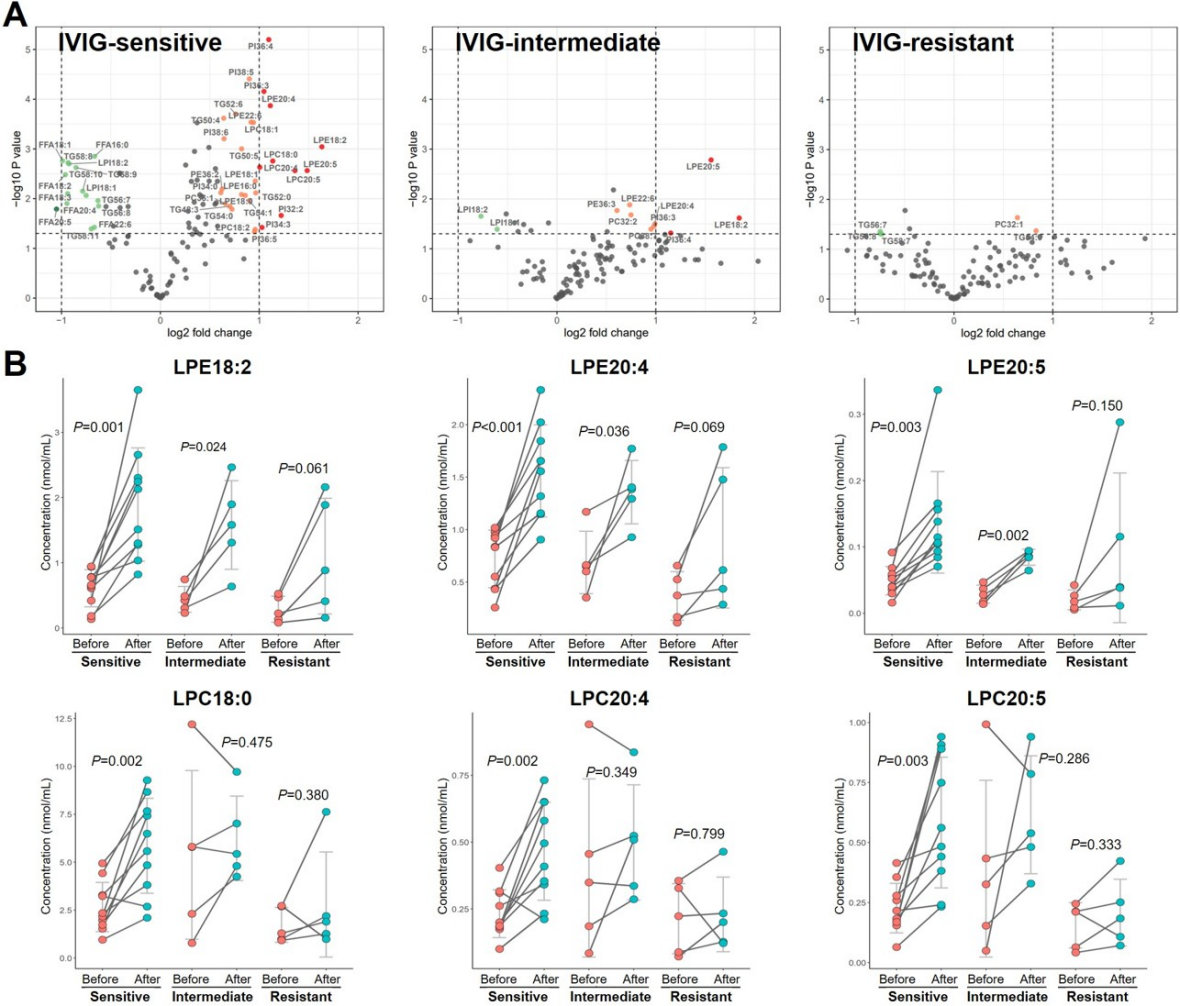
(A) Volcano plots for the lipid changes after treatment in IVIG-sensitive, IVIG-intermediate, and IVIG-resistant groups. The lipid species with a change fold of more than 50% and a *P* value less than 0.05 were considered significant changing features and annotated in color. (B) The concentration of significantly changed LPE and LPC species among the three IVIG-response groups. Scatters in red and cyan stand for the samples before and the samples after treatment, respectively. Solid lines connecting two points indicate the same patient.

**Fig 5:**
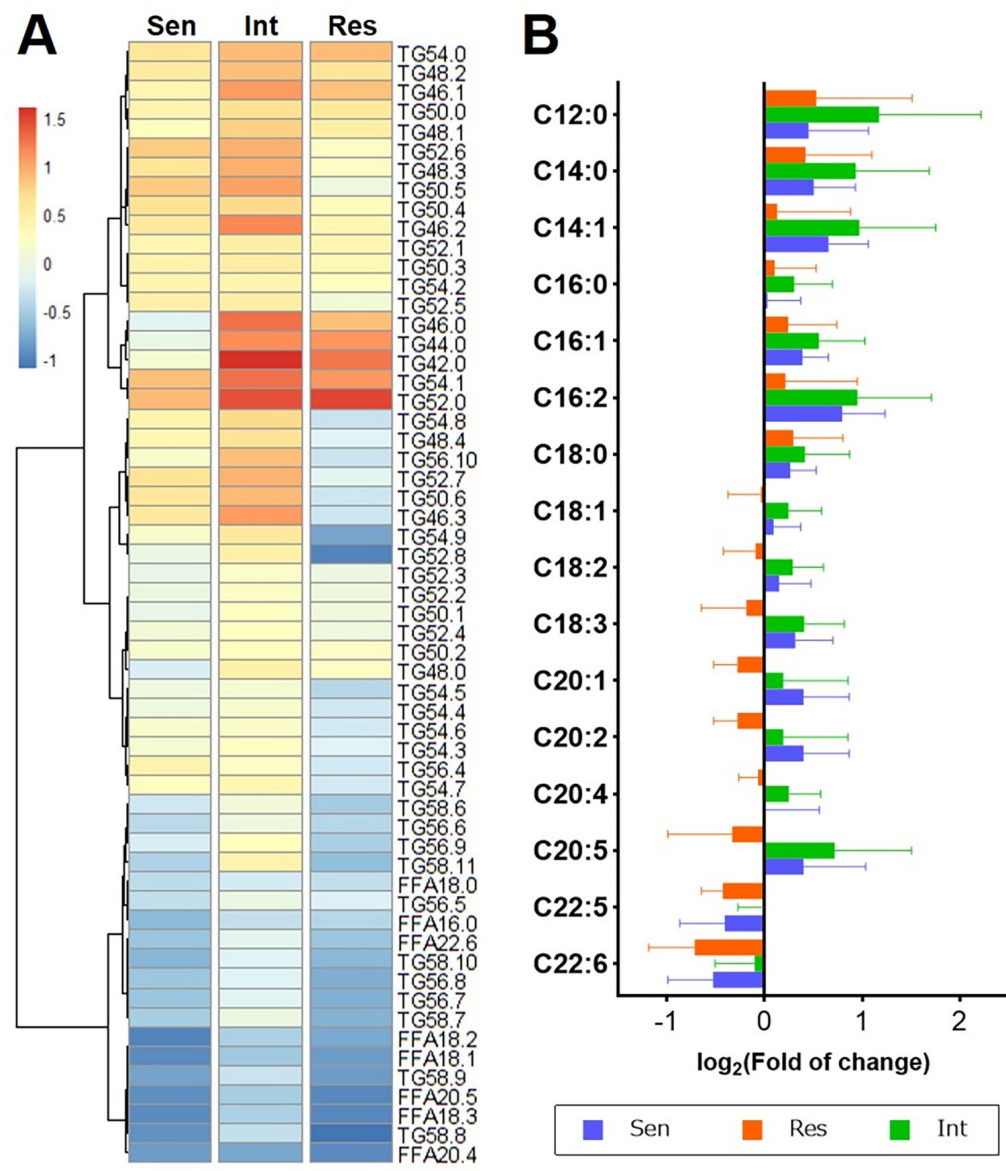
(A) Heatmap for the relative changes of TG and FFA species during KD treatment. (B) Changes of each fatty acyl in TG for Sen (blue), Int (green), and Res (red) groups between the before- and after-treatment groups.

The significant changes in lipids during treatment in the three IVIG-response types are shown in volcano plots (Fig 4A). Notably, LPC and LPE species showed the largest increase among the changed lipids, especially in the Sen group. Interestingly, this trend was weaker in the Int group and even statistically insignificant in the Res group (Fig 4B). On the other side, the decreased lipids mainly consisted of FFA and TG species. However, it is noted that different TG species showed opposite patterns of changes according to their carbon chain length: C48–C54 TG species (e.g., TG50:5) increased, while C56–C58 TG species (e.g., TG58:9) decreased (Fig 5A). Besides, the fatty acyls of TG were compared (Fig 5B) in which the shorter fatty acyls (C12:0 to C18:0) showed similar increasing trends in all the patients, whereas the longer and more unsaturated fatty acyls (C18:1 to C20:5) were increased in Sen and Int groups but decreased in Res group. For instance, for 20:5, the Res group showed an average decrease of 14.5% during treatment, whereas Sen and Int groups showed an increase of 44.5% and 83.7%, respectively. Intriguingly, C22:5 and C22:6 in all the groups expressed reduction.

### 3.4. Lipid variations and their associations with the clinical index after treatment

**Fig 6:**
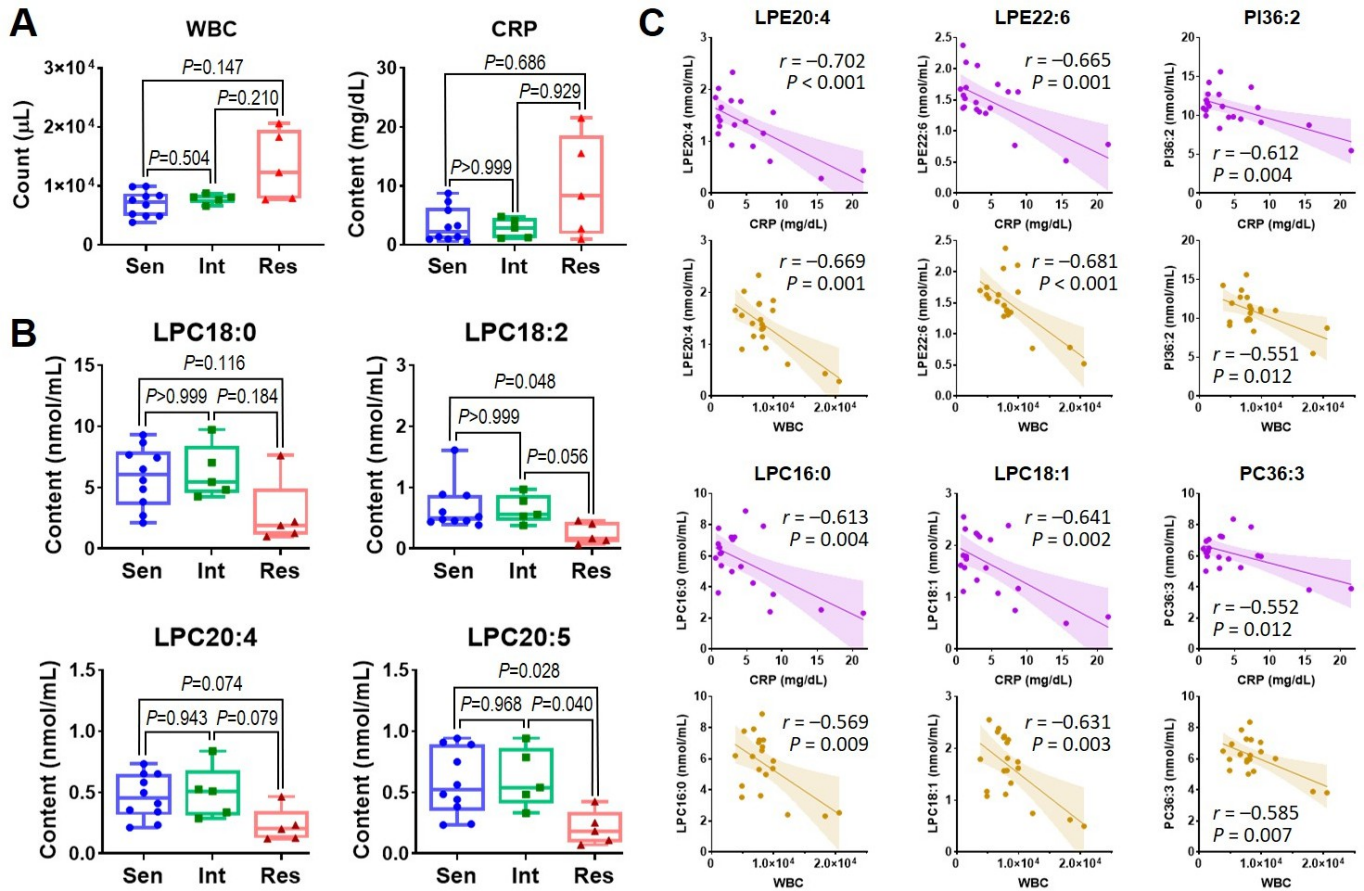
(A) Comparison of WBC and CRP among IVIG-sensitive, IVIG-intermediate, and IVIG-resistant groups after treatment. (B) Comparison of the most variant lipid species among IVIG-sensitive, IVIG-intermediate, and IVIG-resistant groups after treatment. (C) Correlation between the top relevant lipid species (LPE20:4, LPE22:6, PI36:2, LPC16:0, LPC18:1, PC36:3) and clinical features (WBC and CRP).

After treatment, the Res group showed higher WBC counts and CPR levels than the Sen group (*P* = 0.147 and 0.686, respectively) (Fig 6A). While with regard to lipid profile, the top four differentiated species were all LPC: 18:0, 18:2, 20:4, and 20:5, as shown in Fig 6B. Among them, the most significant variation was revealed in LPC20:5, for which the content of the Res group (0.21 ± 0.14 nmol/mL) accounted only 35.7% and 33.8% of Sen (0.58 ± 0.27 nmol/mL, *P* = 0.028) and Int groups (0.62 ± 0.25 nmol/mL, *P* = 0.040), respectively. Moreover, to explore the relationship between lipid characteristics and the inflammatory markers (WBC and CRP in this study), Pearson’s correlation coefficients between every lipid species and these two clinical features were calculated. As a result, all the significant correlations were negative (*r* < −0.4), in which the strongest correlations with WBC and CRP appeared at LPE20:4 (*r* = −0.669 for WBC and −0.702 for CRP) and LPE22:6 (*r* = −0.681 for WBC and −0.665 for CRP), followed by LPC16:0, LPC18:1, PI36:2, and PC36:3 (Fig 6C).

## 4. Discussion

This study provided new evidence of lipidomic characteristics associated with IVIG responsiveness. At present, the widely-accepted diagnostic models for IVIG resistance in KD, including Gunma, Kurume, and Osaka scorings, are primarily based on patient clinical data, such as increased neutrophil counts, decreased platelet counts, hyponatremia, hepatic dysfunction, and elevated CRP levels [11–13]. Other studies used additional indexes, including red blood cell distribution width, percentage of lymphocyte, and total bile acid [27]. Recently, some studies explored the usefulness of transcriptomics to predict response to initial immunoglobulin treatment in KD [28, 29]. While our current work showed that differential lipid profiles could stratify IVIG sensitivity from a different perspective. Since Gunma scoring is known as one of the most widely accepted evaluation systems for IVIG resistance [11], we investigated the relationship between current lipidomic data and the Gunma scores of the patients. There seemed to be only a weak correlation, with the Pearson coefficients ranging from −0.568 (for LPC18:2) to 0.534 (for PC32:1) (S1 Fig). These results suggest that specific lipid species could shed a different light on understanding IVIG-resistant KD. The guidelines of the current diagnosis were reported to achieve both sensitivity and specificity of about 80% [30], whereas some reports claimed these scoring systems with unsatisfied sensitivity (< 45%) [31]. Therefore, more reliable markers are urgently needed. We believe that the combination of lipidomic profiling and clinical data could more accurately stratify IVIG-resistant KD patients who require alternative therapy (Fig 3C); moreover, multiple omics data, such as transcriptomics and proteomics, could possibly be connected to lipidomics to find new insights into IVIG resistance.

Amongst the discovered lipids, lysophospholipids (LPE and LPC) were the most variable species. They were all low levels of lipids in the Res group both before and after IVIG treatment, while they were markedly increased in Sen and Int groups following IVIG treatment. These membrane-derived lysophospholipids are considered critical signaling molecules that participate in the regulation of inflammatory response [32], but their specific roles in inflammation are still controversial. On one side, lysophospholipids were reported as important mediators contributing to the development and progression of atherosclerosis by regulating inflammatory events in cardiovascular disease [33]. On the other side, serum LPC was decreased by elevated inflammatory status, such as insulin resistance, nonalcoholic steatohepatitis (NASH), and cancer-involved inflammation, where negative correlations were found between LPC and inflammatory markers (CRP and TNF-α) [34–36]. In our current study, Res KD exhibited lower levels of lysophospholipids, which could be supposedly suppressed by persistent inflammation in the bloodstream [1]. As phospholipase A2 superfamily are vital enzymes to form lysophospholipids [37], it is presumed that the altered enzyme activities may cause the depletion of LPE and LPC in the Res group following IVIG treatment. Nevertheless, the precise mechanism remains to be investigated.

In addition, TG species were found to express distinctive changes along with IVIG-response types during the treatment. It is known that TG profiling plays a vital role in cardiovascular diseases, including type II diabetes [38], coronary artery diseases [39], and even cardiovascular death [40]. Besides, the long-chain unsaturated fatty acyls are considered to slow down intra-arterial occlusion and platelet aggregation, contributing to a lower risk for atherosclerosis and thrombus [41]. In our results, only the Res group showed a negative change in long-chain unsaturated fatty acyl levels, which might be due to the distinguished glycerolipid remodeling process. According to IVIG responsiveness, the different genetic and enzymatic reactions could trigger the remodeling of glycerolipids and transfer the fatty acyls, finally resulting in the specific alteration of the TG profile in Res patients during treatment. Another notable finding was that although the different IVIG-response groups shared the same changing trends of lipidomic characteristics during treatment, the changing significance (intensity of variation) among groups followed this order: Sen > Int > Res. These results indicate that the more IVIG resistance that patients show, the poorer lipid metabolism responds in KD. Therefore, the revealed lipidomic data might propose the chemical basis of lipid molecules involved in KD inflammation.

There were several limitations in this study. Firstly, we only investigated KD patients with different IVIG responsiveness but did not include healthy subjects; the sample size also needed to be extended to establish more reliable and robust diagnosis/prediction models. Further efforts would combine a larger range of subjects composed of healthy status, regions, IVIG responsiveness, and other epidemiological factors. Secondly, the genes and enzymes related to lipidomic characteristics, which would reveal the upstream changes of regulation, were not investigated. While this study focused on the characterization of lipidomic profiles associated with IVIG responsiveness, further investigations into the underlying genetic and enzymatic changes are warranted to fully understand the mechanisms of IVIG resistance in KD. Integrating expression profiles with lipidomics data could provide more insights into the pathophysiology of KD. In future works, multi-omics studies should be integrated to understand the underlying mechanisms that mediate the different phenotypes. Thirdly, the present analytical procedure omitted some polar lipids, such as glycolipids and sphingolipids, which might also be involved in the pathophysiology of KD [42]. Therefore, the measurement of hydrophilic molecules, including not only polar lipids but also small metabolites (e.g., amino acids, organic acids, nucleotides), should be carried out in the future.

## 5. Conclusion

In summary, this study focused on the comparison among IVIG responsiveness of KD patients. The LC/MS-based lipidomic data revealed that lipid profiles expressed similar patterns between the IVIG-sensitive and intermediate groups, but significant differences in the IVIG-resistant group before and after IVIG treatment. Lysophospholipids LPC and LPE species were able to stratify KD patients in IVIG-responsiveness, which can become potential diagnostic markers before treatment, and, if combined with the current scoring systems, could enhance the IVIG-resistant prediction. The strong correlations of lysophospholipids and the inflammatory markers (WBC and CRP) after treatment suggested they can be helpful in knowing persistent inflammation status even after being treated, which could lead to KD sequelae risks. Moreover, the lysophospholipids formation and FFA acylation were supposed to be involved in the phospholipid remodeling process in IVIG-resistant patients. These distinctive changes during treatment would not only contribute to the chemical basis concerning lipid metabolism but also help to elucidate the mechanism of IVIG resistance in KD. Future studies integrating multiple omics data and larger sample sizes are needed to fully elucidate the underlying mechanisms of IVIG resistance in KD and to translate these findings into clinical practice.

## Supporting information

S1 Table Clinical features of the KD patients. (DOCX)

S2 Table LC elution gradient in ESI-positive and -negative modes. (DOCX)

S3 Table Annotation and identification of the lipid species according to the LC/MS data. (DOCX)

S4 Table Semi-quantitative concentration of each lipid species. (CSV)

S5 Table Gunma scoring, lipid scoring, and the combined scoring for the investigated patients. (DOCX)

S1 Fig Correlation between Gunma score and content of the potential lipid markers. (DOCX)
