# Supplementary material for: Distinctive serum lipidomic profile of IVIG-resistant Kawasaki disease children before and after treatment
Source: PLoS One. 2023 Mar 29;18(3):e0283710. doi: 10.1371/journal.pone.0283710 (PMC10057782; doi:10.1371/journal.pone.0283710)
Supplement: S1 Table — (DOCX) [file pone.0283710.s001.docx]

**S1 Table** Clinical Features of the KD patients

| Parameters  N | IVIG-sensitive  10 | IVIG-intermediate  5 | IVIG-resistant  5 |
| --- | --- | --- | --- |
| Male : Female (%) | 7(70%) : 3(30%) | 0(0%) : 5(100%) | 4(80%) : 1(20%) |
| Age (Months) | 34.9 ± 20.3 | 45.0 ± 23.2 | 38.8 ± 14.5 |
| Onset : Recurrence (%) | 9(90%) : 1(10%) | 4(80%) : 1(20%) | 4(80%) : 1(20%) |
| White blood cell  before treatment (count/µL) | 13783 ± 4349 | 15740 ± 3173 | 15120 ± 3604 |
| White blood cell  after treatment (count/µL) | 6958 ± 2178 ^b^ | 7850 ± 807 ^b^ | 13348 ± 5904 ^a^ |
| C-reactive protein  before treatment (mg/dL) | 10.04 ± 6.59 | 9.26 ± 5.52 | 9.90 ± 3.67 |
| C-reactive protein  after treatment (mg/dL) | 3.35 ± 2.95 ^b^ | 2.84 ± 1.68 ^b^ | 9.82 ± 8.67 ^a^ |
| Gunma score | 2.5 ± 2.0 ^b^ | 2.4 ± 2.3 ^b^ | 6.4 ± 1.9 ^a^ |

a,b: Values of the same row with different letters differ at *P* < 0.05, calculated by one-way ANOVA with Tukey’s post hoc test. Data were expressed as means ± SD.
