# Supplementary material for: Distinctive serum lipidomic profile of IVIG-resistant Kawasaki disease children before and after treatment
Source: PLoS One. 2023 Mar 29;18(3):e0283710. doi: 10.1371/journal.pone.0283710 (PMC10057782; doi:10.1371/journal.pone.0283710)
Supplement: S2 Table — (DOCX) [file pone.0283710.s002.docx]

**S1 Table** LC elution gradient in ESI-positive and -negative modes

| Mode | Mobile phase /% | Time /min | | | | | |
| --- | --- | --- | --- | --- | --- | --- | --- |
|  |  | 0 | 1 | 10 | 19 | 20 | 23 |
| ESI-Positive | A (10 mM aqueous ammonium acetate) | 4 | 4 | 2 | 2 | 4 | 4 |
|  | B (isopropanol) | 6 | 6 | 83 | 83 | 6 | 6 |
|  | C (methanol) | 90 | 90 | 15 | 15 | 90 | 90 |
| ESI-Negative | A (10 mM aqueous ammonium acetate) | 25 | 5 | 5 | 0 | 25 | 25 |
|  | B (isopropanol) | 40 | 60 | 60 | 65 | 40 | 40 |
|  | C (methanol) | 35 | 35 | 35 | 35 | 35 | 35 |
