# Supplementary material for: Distinctive serum lipidomic profile of IVIG-resistant Kawasaki disease children before and after treatment
Source: PLoS One. 2023 Mar 29;18(3):e0283710. doi: 10.1371/journal.pone.0283710 (PMC10057782; doi:10.1371/journal.pone.0283710)
Supplement: S3 Table — (DOCX) [file pone.0283710.s003.docx]

**S2 Table** Annotation and identification of the lipid species according to the LC/MS data

| Lipid species | Formula | Diagnostic ion | Retention time | Calc. *m/z* | Exp. *m/z* | Δppm | Fragment ion(s) | Fatty acyl composition |
| --- | --- | --- | --- | --- | --- | --- | --- | --- |
| TG42:0 | C_45_H_86_O_6_ | [M + NH_4_]^+^ | 13.38 | 740.6763 | 740.6767 | 0.54 | 495  467,495,523 | 14:0/14:0/14:0  12:0/16:0/14:0 |
| TG44:0 | C_47_H_90_O_6_ | [M + NH_4_]^+^ | 13.80 | 768.7076 | 768.7079 | 0.39 | 495,523  495,551 | 14:0/16:0/14:0  12:0/16:0/16:0 |
| TG46:0 | C_49_H_94_O_6_ | [M + NH_4_]^+^ | 14.19 | 796.7389 | 796.7396 | 0.88 | 523,551  495,523,579 | 14:0/16:0/16:0  12:0/18:0/16:0 |
| TG46:1 | C_49_H_92_O_6_ | [M + NH_4_]^+^ | 13.86 | 794.7232 | 794.7238 | 0.75 | 549,523,521  577,521,495 | 14:0/16:1/16:0  12:0/18:1/16:0 |
| TG46:2 | C_49_H_90_O_6_ | [M + NH_4_]^+^ | 13.56 | 792.7076 | 792.7078 | 0.25 | 549,521,519  575,521,493 | 14:1/16:1/16:0  12:0/16:1/18:1 |
| TG46:3 | C_49_H_88_O_6_ | [M + NH_4_]^+^ | 13.28 | 790.6919 | 790.6923 | 0.51 | 547,519  547,545,493 | 14:1/16:1/16:1  14:0/14:1/18:2 |
| TG48:0 | C_51_H_98_O_6_ | [M + NH_4_]^+^ | 14.54 | 824.7702 | 824.7709 | 0.85 | 551  523,551,579 | 16:0/16/0/16:0 |
| TG48:1 | C_51_H_96_O_6_ | [M + NH_4_]^+^ | 14.23 | 822.7545 | 822.7551 | 0.73 | 549,551  523,549,577 | 16:0/16:1/16:0  14:0/18:1/16:0 |
| TG48:2 | C_51_H_94_O_6_ | [M + NH_4_]^+^ | 13.93 | 820.7389 | 820.7386 | −0.37 | 547,549  521,549,575 | 16:0/16:1/16:1  14:0/18:1/16:1 |
| TG48:3 | C_51_H_92_O_6_ | [M + NH_4_]^+^ | 13.63 | 818.7232 | 818.7231 | −0.12 | 547  521,547,573  519,521,601 | 16:1/16:1/16:1  14:0/18:2/16:1  12:0/18:1/18:2 |
| TG48:4 | C_51_H_90_O_6_ | [M + NH_4_]^+^ | 13.35 | 816.7076 | 816.7065 | −1.35 | N/D | N/D |
| TG50:0 | C_53_H_102_O_6_ | [M + NH_4_]^+^ | 14.94 | 852.8015 | 852.8019 | 0.47 | 551,579 | 16:0/18:0/16:0 |
| TG50:1 | C_53_H_100_O_6_ | [M + NH_4_]^+^ | 14.58 | 850.7858 | 850.7862 | 0.47 | 551,577 | 16:0/18:1/16:0 |
| TG50:2 | C_53_H_98_O_6_ | [M + NH_4_]^+^ | 14.30 | 848.7702 | 848.7698 | −0.47 | 549,575,577 | 16:0/18:1/16:1 |
| TG50:3 | C_53_H_96_O_6_ | [M + NH_4_]^+^ | 14.01 | 846.7545 | 846.7538 | −0.83 | 547,575  547,549,601  549,573,575 | 16:1/18:1/16:1  14:0/18:2/18:1  16:0/16:1/18:2 |
| TG50:4 | C_53_H_94_O_6_ | [M + NH_4_]^+^ | 13.73 | 844.7389 | 844.7385 | −0.47 | 547,573  547,599  545,547,601 | 16:1/18:2/16:1  14:0/18:2/18:2  14:1/18:2/18:1 |
| TG50:5 | C_53_H_92_O_6_ | [M + NH_4_]^+^ | 13.47 | 842.7232 | 842.7232 | 0.00 | 545,547,597  543,547,599  545,599 | 14:0/18:3/18:2  14:1/18:3/18:1  14:0/18:2/18:2 |
| TG50:6 | C_53_H_90_O_6_ | [M + NH_4_]^+^ | 13.24 | 840.7076 | 840.7067 | −1.07 | N/D | N/D |
| TG52:0 | C_55_H_106_O_6_ | [M + NH_4_]^+^ | 15.41 | 880.8328 | 880.8331 | 0.34 | 579,607 | 16:0/18:0/18:0 |
| TG52:1 | C_55_H_104_O_6_ | [M + NH_4_]^+^ | 14.98 | 878.8171 | 878.8173 | 0.23 | 577,579,605 | 16:0/18:1/18:0 |
| TG52:2 | C_55_H_102_O_6_ | [M + NH_4_]^+^ | 14.66 | 876.8015 | 876.8010 | −0.57 | 577,603  575,579,603 | 16:0/18:1/18:1  16:0/18:0/18:2 |
| TG52:3 | C_55_H_100_O_6_ | [M + NH_4_]^+^ | 14.40 | 874.7858 | 874.7856 | −0.23 | 575,577,601 | 16:0/18:1/18:2 |
| TG52:4 | C_55_H_98_O_6_ | [M + NH_4_]^+^ | 14.12 | 872.7702 | 872.7700 | −0.23 | 573,575,601  575,599 | 16:1/18:2/18:1  16:0/18:2/18:2 |
| TG52:5 | C_55_H_96_O_6_ | [M + NH_4_]^+^ | 13.86 | 870.7545 | 870.7546 | 0.11 | 573,599 | 16:1/18:2/18:2 |
| TG52:6 | C_55_H_94_O_6_ | [M + NH_4_]^+^ | 13.54 | 868.7389 | 868.7386 | −0.35 | 571,573,597  571,599  573,595  547,571,623 | 16:1/18:3/18:2  16:2/18:2/18:2  16:0/18:3/18:3  14:0/18:2/20:4 |
| TG52:7 | C_55_H_92_O_6_ | [M + NH_4_]^+^ | 13.45 | 866.7232 | 866.7229 | −0.35 | N/D | N/D |
| TG52:8 | C_55_H_90_O_6_ | [M + NH_4_]^+^ | 13.14 | 864.7076 | 864.7072 | −0.46 | N/D | N/D |
| TG54:0 | C_57_H_110_O_6_ | [M + NH_4_]^+^ | 15.86 | 908.8641 | 908.8644 | 0.33 | 607 | 18:0/18:0/18:0 |
| TG54:1 | C_57_H_108_O_6_ | [M + NH_4_]^+^ | 15.44 | 906.8484 | 906.8485 | 0.11 | 605,607 | 18:0/18:1/18:0 |
| TG54:2 | C_57_H_106_O_6_ | [M + NH_4_]^+^ | 15.07 | 904.8328 | 904.8324 | −0.44 | 603,605 | 18:0/18:1/18:1 |
| TG54:3 | C_57_H_104_O_6_ | [M + NH_4_]^+^ | 14.79 | 902.8171 | 902.8173 | 0.22 | 603 | 18:1/18:1/18:1 |
| TG54:4 | C_57_H_102_O_6_ | [M + NH_4_]^+^ | 14.51 | 900.8015 | 900.8003 | −1.33 | 601,603 | 18:1/18:2/18:1 |
| TG54:5 | C_57_H_100_O_6_ | [M + NH_4_]^+^ | 14.19 | 898.7858 | 898.7852 | −0.67 | 599,601 | 18:1/18:2/18:2 |
| TG54:6 | C_57_H_98_O_6_ | [M + NH_4_]^+^ | 13.93 | 896.7702 | 896.7688 | −1.56 | 597,599,601 | 18:1/18:3/18:2 |
| TG54:7 | C_57_H_96_O_6_ | [M + NH_4_]^+^ | 13.82 | 894.7545 | 894.7535 | −1.12 | 597,599 | 18:2/18:3/18:2 |
| TG54:8 | C_57_H_94_O_6_ | [M + NH_4_]^+^ | 13.58 | 892.7389 | 892.7385 | −0.45 | 547,595,647  573,595,621 | 14:0/22:6/18:2  16:1/18:2/20:5 |
| TG54:9 | C_57_H_92_O_6_ | [M + NH_4_]^+^ | 13.32 | 890.7232 | 890.7227 | −0.56 | N/D | N/D |
| TG56:4 | C_59_H_106_O_6_ | [M + NH_4_]^+^ | 14.84 | 928.8328 | 928.8322 | −0.65 | 603,629  601,629,631 | 18:1/20:2/18:1  18:1/20:1/18:2 |
| TG56:5 | C_59_H_104_O_6_ | [M + NH_4_]^+^ | 14.73 | 926.8171 | 926.8160 | −1.19 | 605,625,627 | 18:1/20:4/18:0 |
| TG56:6 | C_59_H_102_O_6_ | [M + NH_4_]^+^ | 14.51 | 924.8015 | 924.7997 | −1.95 | 577,625,651 | 16:0/18:1/22:5 |
| TG56:7 | C_59_H_100_O_6_ | [M + NH_4_]^+^ | 14.24 | 922.7858 | 922.7848 | −1.08 | 577,623,649 | 16:0/22:6/18:1 |
| TG56:8 | C_59_H_98_O_6_ | [M + NH_4_]^+^ | 13.98 | 920.7702 | 920.7693 | −0.98 | 575,623,647 | 16:0/22:6/18:2 |
| TG56:9 | C_59_H_96_O_6_ | [M + NH_4_]^+^ | 13.65 | 918.7545 | 918.7540 | −0.54 | 573,621,647 | 16:1/22:6/18:2 |
| TG56:10 | C_59_H_94_O_6_ | [M + NH_4_]^+^ | 13.38 | 916.7389 | 916.7380 | −0.98 | 571,621,645 | 16:1/22:6/18:3 |
| TG58:6 | C_61_H_106_O_6_ | [M + NH_4_]^+^ | 14.80 | 952.8328 | 952.8325 | −0.31 | N/D | N/D |
| TG58:7 | C_61_H_104_O_6_ | [M + NH_4_]^+^ | 14.52 | 950.8171 | 950.8164 | −0.74 | N/D | N/D |
| TG58:8 | C_61_H_102_O_6_ | [M + NH_4_]^+^ | 14.30 | 948.8015 | 948.8005 | −1.05 | 603,649 | 18:1/22:6/18:1 |
| TG58:9 | C_61_H_100_O_6_ | [M + NH_4_]^+^ | 14.05 | 946.7858 | 946.7852 | −0.63 | 601,647,649 | 18:1/22:6/18:2 |
| TG58:10 | C_61_H_98_O_6_ | [M + NH_4_]^+^ | 13.77 | 944.7702 | 944.7703 | 0.11 | 599,647 | 18:2/22:6/18:2 |
| TG58:11 | C_61_H_96_O_6_ | [M + NH_4_]^+^ | 13.52 | 942.7545 | 942.7543 | −0.21 | N/D | N/D |
| FFA16:0 | C_16_H_32_O_2_ | [M − H]^−^ | 11.53 | 255.2330 | 255.2331 | 0.39 | N/A | 16:0 |
| FFA18:0 | C_18_H_36_O_2_ | [M − H]^−^ | 13.34 | 283.2643 | 283.2640 | −1.06 | N/A | 18:0 |
| FFA18:1 | C_18_H_34_O_2_ | [M − H]^−^ | 12.07 | 281.2486 | 281.2488 | 0.71 | N/A | 18:1 |
| FFA18:2 | C_18_H_32_O_2_ | [M − H]^−^ | 10.78 | 279.2330 | 279.2332 | 0.72 | N/A | 18:2 |
| FFA18:3 | C_18_H_30_O_2_ | [M − H]^−^ | 9.49 | 277.2173 | 277.2177 | 1.44 | N/A | 18:3 |
| FFA20:4 | C_20_H_32_O_2_ | [M − H]^−^ | 10.76 | 303.2330 | 303.2328 | −0.66 | N/A | 20:4 |
| FFA20:5 | C_20_H_30_O_2_ | [M − H]^−^ | 9.45 | 301.2173 | 301.2172 | −0.33 | N/A | 20:5 |
| FFA22:6 | C_22_H_32_O_2_ | [M − H]^−^ | 10.36 | 327.2330 | 327.2334 | 1.22 | N/A | 22:6 |
| PC32:0 | C_40_H_80_O_8_NP | [M + H]^+^ | 9.39 | 734.5694 | 734.5701 | 0.95 | 478 | 16:0/16:0 |
| PC32:1 | C_40_H_78_O_8_NP | [M + H]^+^ | 8.68 | 732.5538 | 732.5536 | −0.27 | 476,478 | 16:0/16:1 |
| PC32:2 | C_40_H_76_O_8_NP | [M + H]^+^ | 8.24 | 730.5381 | 730.5378 | −0.41 | 450,502 | 14:0/18:2 |
| PC34:1 | C_42_H_82_O_8_NP | [M + H]^+^ | 9.56 | 760.5851 | 760.5848 | −0.39 | 478,504 | 16:0/18:1 |
| PC34:2 | C_42_H_80_O_8_NP | [M + H]^+^ | 9.15 | 758.5694 | 758.5695 | 0.13 | 478,502 | 16:0/18:2 |
| PC34:3 | C_42_H_78_O_8_NP | [M + H]^+^ | 8.58 | 756.5538 | 756.5535 | −0.40 | 478,500 | 16:0/18:3 |
| PC34:4 | C_42_H_76_O_8_NP | [M + H]^+^ | 8.15 | 754.5381 | 754.5380 | −0.13 | N/D | N/D |
| PC36:1 | C_44_H_86_O_8_NP | [M + H]^+^ | 10.98 | 788.6164 | 788.6158 | −0.76 | 504,506 | 18:0/18:1 |
| PC36:2 | C_44_H_84_O_8_NP | [M + H]^+^ | 9.96 | 786.6007 | 786.6005 | −0.25 | 502,506  504 | 18:0/18:2  18:1/18:1 |
| PC36:3 | C_44_H_82_O_8_NP | [M + H]^+^ | 9.18 | 784.5851 | 784.5839 | −1.53 | 502,504 | 18:1/18:2 |
| PC36:4 | C_44_H_80_O_8_NP | [M + H]^+^ | 8.75 | 782.5694 | 782.5698 | 0.51 | 478,526 | 16:0/20:4 |
| PC36:5 | C_44_H_78_O_8_NP | [M + H]^+^ | 8.43 | 780.5538 | 780.5534 | −0.51 | 478,524 | 16:0/20:5 |
| PC36:6 | C_44_H_76_O_8_NP | [M + H]^+^ | 8.12 | 778.5381 | 778.5380 | −0.13 | 450,550 | 14:0/22:6 |
| PC38:4 | C_46_H_84_O_8_NP | [M + H]^+^ | 9.75 | 810.6007 | 810.6005 | −0.25 | 506,526 | 18:0/20:4 |
| PC38:5 | C_46_H_82_O_8_NP | [M + H]^+^ | 9.15 | 808.5851 | 808.5839 | −1.48 | 504,526  478,552 | 18:1/20:4  16:0/22:5 |
| PC38:6 | C_46_H_80_O_8_NP | [M + H]^+^ | 8.70 | 806.5694 | 806.5688 | −0.74 | 478,550 | 16:0/22:6 |
| PC38:7 | C_46_H_78_O_8_NP | [M + H]^+^ | 8.22 | 804.5538 | 804.5532 | −0.75 | N/D | N/D |
| PC40:6 | C_48_H_84_O_8_NP | [M + H]^+^ | 9.66 | 834.6007 | 834.6008 | 0.12 | 506,550 | 18:0/22:6 |
| PE34:1 | C_39_H_76_O_8_NP | [M − H]^−^ | 17.22 | 716.5236 | 716.5253 | 2.37 | 255,281,452 | 16:0/18:1 |
| PE34:2 | C_39_H_74_O_8_NP | [M − H]^−^ | 16.77 | 714.5079 | 714.5098 | 2.66 | 255,279,452 | 16:0/18:2 |
| PE36:2 | C_41_H_78_O_8_NP | [M − H]^−^ | 17.49 | 742.5392 | 742.5413 | 2.83 | 279,283,480 | 18:0/18:2 |
| PE36:3 | C_41_H_76_O_8_NP | [M − H]^−^ | 17.08 | 740.5236 | 740.5255 | 2.57 | N/D | N/D |
| PE36:4 | C_41_H_74_O_8_NP | [M − H]^−^ | 16.78 | 738.5079 | 738.5097 | 2.44 | 255,303,452 | 16:0/20:4 |
| PE36:5 | C_41_H_72_O_8_NP | [M − H]^−^ | 16.40 | 736.4923 | 736.4937 | 1.90 | N/D | N/D |
| PE38:4 | C_43_H_78_O_8_NP | [M − H]^−^ | 17.58 | 766.5392 | 766.5409 | 2.22 | 283,303,480 | 18:0/20:4 |
| PE38:5 | C_43_H_76_O_8_NP | [M − H]^−^ | 17.17 | 764.5236 | 764.5253 | 2.22 | N/D | N/D |
| PE38:6 | C_43_H_74_O_8_NP | [M − H]^−^ | 16.77 | 762.5079 | 762.5088 | 1.18 | 255,327,452 | 16:0/22:6 |
| PI32:0 | C_41_H_79_O_13_P | [M − H]^−^ | 14.17 | 809.5186 | 809.5183 | −0.37 | 255,391,553 | 16:0/16:0 |
| PI32:1 | C_41_H_77_O_13_P | [M − H]^−^ | 13.65 | 807.5029 | 807.5035 | 0.74 | 253,255,391,553 | 16:0/16:1 |
| PI32:2 | C_41_H_75_O_13_P | [M − H]^−^ | 13.14 | 805.4873 | 805.4871 | −0.25 | 253,389,551 | 16:1/16:1 |
| PI34:0 | C_43_H_83_O_13_P | [M − H]^−^ | 14.86 | 837.5499 | 837.5501 | 0.24 | 255,283,391,553 | 16:0/18:0 |
| PI34:1 | C_43_H_81_O_13_P | [M − H]^−^ | 14.37 | 835.5342 | 835.5352 | 1.20 | 255,281,419,553 | 16:0/18:1 |
| PI34:2 | C_43_H_79_O_13_P | [M − H]^−^ | 13.93 | 833.5186 | 833.5187 | 0.12 | 255,279,415,553 | 16:0/18:2 |
| PI34:3 | C_43_H_77_O_13_P | [M − H]^−^ | 13.53 | 831.5029 | 831.5029 | 0.00 | N/D | N/D |
| PI36:1 | C_45_H_85_O_13_P | [M − H]^−^ | 15.05 | 863.5655 | 863.5654 | −0.12 | 281,283,419,581 | 18:0/18:1 |
| PI36:2 | C_45_H_83_O_13_P | [M − H]^−^ | 14.60 | 861.5499 | 861.5497 | −0.23 | 281,417,579 | 18:1/18:1 |
| PI36:3 | C_45_H_81_O_13_P | [M − H]^−^ | 14.11 | 859.5342 | 859.5345 | 0.35 | N/D | N/D |
| PI36:4 | C_45_H_79_O_13_P | [M − H]^−^ | 13.99 | 857.5186 | 857.5187 | 0.12 | 255,303,391,553 | 16:0/20:4 |
| PI36:5 | C_45_H_77_O_13_P | [M − H]^−^ | 13.60 | 855.5029 | 855.5032 | 0.35 | N/D | N/D |
| PI38:4 | C_47_H_83_O_13_P | [M − H]^−^ | 14.71 | 885.5499 | 885.5498 | −0.11 | 283,303,419,581 | 18:0/20:4 |
| PI38:5 | C_47_H_81_O_13_P | [M − H]^−^ | 14.35 | 883.5342 | 883.5349 | 0.79 | 281,303,417,579 | 18:1/20:4 |
| PI38:6 | C_47_H_79_O_13_P | [M − H]^−^ | 13.97 | 881.5186 | 881.5181 | −0.57 | N/D | N/D |
| PI40:6 | C_49_H_83_O_13_P | [M − H]^−^ | 14.69 | 909.5499 | 909.5485 | −1.54 | 283,327,419,581 | 18:0/22:6 |
| LPC16:0 | C_24_H_50_O_7_NP | [M + H]^+^ | 3.49 | 496.3398 | 496.3400 | 0.40 | N/A | 16:0 |
| LPC18:0 | C_26_H_54_O_7_NP | [M + H]^+^ | 4.83 | 524.3711 | 524.3705 | −1.14 | N/A | 18:0 |
| LPC18:1 | C_26_H_52_O_7_NP | [M + H]^+^ | 3.43 | 522.3554 | 522.3552 | −0.38 | N/A | 18:1 |
| LPC18:2 | C_26_H_50_O_7_NP | [M + H]^+^ | 3.13 | 520.3398 | 520.3394 | −0.77 | N/A | 18:2 |
| LPC20:4 | C_28_H_50_O_7_NP | [M + H]^+^ | 3.37 | 544.3398 | 544.3391 | −1.29 | N/A | 20:4 |
| LPC20:5 | C_28_H_48_O_7_NP | [M + H]^+^ | 3.00 | 542.3241 | 542.3230 | −2.03 | N/A | 20:5 |
| LPE16:0 | C_21_H_44_O_7_NP | [M − H]^−^ | 9.89 | 452.2783 | 452.2785 | 0.44 | N/A | 16:0 |
| LPE18:0 | C_23_H_48_O_7_NP | [M − H]^−^ | 11.64 | 480.3096 | 480.3098 | 0.42 | N/A | 18:0 |
| LPE18:1 | C_23_H_46_O_7_NP | [M − H]^−^ | 10.40 | 478.2939 | 478.2937 | −0.42 | N/A | 18:1 |
| LPE18:2 | C_23_H_44_O_7_NP | [M − H]^−^ | 9.25 | 476.2783 | 476.2782 | −0.21 | N/A | 18:2 |
| LPE20:4 | C_25_H_44_O_7_NP | [M − H]^−^ | 9.31 | 500.2783 | 500.2780 | −0.60 | N/A | 20:4 |
| LPE20:5 | C_25_H_42_O_7_NP | [M − H]^−^ | 8.27 | 498.2626 | 498.2629 | 0.60 | N/A | 20:5 |
| LPE22:6 | C_27_H_44_O_7_NP | [M − H]^−^ | 9.31 | 524.2783 | 524.2786 | 0.57 | N/A | 22:6 |
| LPI16:0 | C_25_H_49_O_12_P | [M − H]^−^ | 7.28 | 571.2889 | 571.2888 | −0.18 | N/A | 16:0 |
| LPI18:0 | C_27_H_53_O_12_P | [M − H]^−^ | 9.11 | 599.3202 | 599.3209 | 1.17 | N/A | 18:0 |
| LPI18:1 | C_27_H_51_O_12_P | [M − H]^−^ | 7.77 | 597.3045 | 597.3046 | 0.17 | N/A | 18:1 |
| LPI18:2 | C_27_H_49_O_12_P | [M − H]^−^ | 6.54 | 595.2889 | 595.2891 | 0.34 | N/A | 18:2 |
| LPI20:4 | C_29_H_49_O_12_P | [M − H]^−^ | 6.62 | 619.2889 | 619.2889 | 0.00 | N/A | 20:4 |

N/A: Not available (the lipid species contained only one fatty acyl, thus identical).

N/D: Not identified (we could not get the MS/MS data due to the low intensity).

TG, triacylglycerol; FFA, free fatty acid; PC, phosphatidylcholine; PE, phosphatidylethanolamine; PI, phosphatidylinositol; LPC, lysophosphatidylcholine; LPE, lysophosphatidylethanolamine; LPI, lysophosphatidylinositol.
