# Supplementary material for: Distinctive serum lipidomic profile of IVIG-resistant Kawasaki disease children before and after treatment
Source: PLoS One. 2023 Mar 29;18(3):e0283710. doi: 10.1371/journal.pone.0283710 (PMC10057782; doi:10.1371/journal.pone.0283710)
Supplement: S5 Table — (DOCX) [file pone.0283710.s005.docx]

**S4 Table** Gunma scoring, lipid scoring, and the combined scoring for the investigated patients

| Patient | Response  to IVIG | Gunma  score | The concentration of selected lipid species (nmol/mL)  (Score based on criteria, score = 1 when fitting the criteria) | | | | | | Lipid  score | Combined  score |
| --- | --- | --- | --- | --- | --- | --- | --- | --- | --- | --- |
|  |  |  | LPC18:2  (≤0.1736) | LPE20:5  (≤0.0267) | LPE20:4  (≤0.5267) | PE34:1  (≥0.2670) | LPE18:1  (≤0.6716) | PC32:1  (≥0.5243) |  |  |
| 1 | Resistant | 5 | 0.0722  (1) | 0.0053  (1) | 0.1142  (1) | 0.2670  (1) | 0.2026  (1) | 0.5332  (1) | 6 | 11 |
| 2 | Intermediate | 1 | 1.1212  (0) | 0.0177  (1) | 0.6502  (0) | 0.1430  (0) | 0.5620  (1) | 0.3438  (0) | 2 | 3 |
| 3 | Resistant | 4 | 0.0635  (1) | 0.0087  (1) | 0.1689  (1) | 0.8720  (1) | 0.3559  (1) | 0.7595  (1) | 6 | 10 |
| 4 | Sensitive | 1 | 0.4710  (0) | 0.0407  (0) | 0.9788  (0) | 0.1110  (0) | 0.8223  (0) | 0.4092  (0) | 0 | 1 |
| 5 | Sensitive | 1 | 0.4102  (0) | 0.0563  (0) | 0.8389  (0) | 0.3860  (1) | 1.3073  (0) | 0.2357  (0) | 1 | 2 |
| 6 | Sensitive | 1 | 0.3913  (0) | 0.0916  (0) | 0.5531  (0) | 0.1400  (0) | 0.7214  (0) | 0.3699  (0) | 0 | 1 |
| 7 | Sensitive | 3 | 0.2126  (0) | 0.0359  (0) | 0.9428  (0) | 0.1840  (0) | 0.6178  (1) | 0.5824  (1) | 2 | 5 |
| 8 | Sensitive | 4 | 0.2497  (0) | 0.0682  (0) | 0.9205  (0) | 0.4930  (1) | 0.8811  (0) | 0.4636  (0) | 1 | 5 |
| 9 | Resistant | 9 | 0.2545  (0) | 0.0426  (0) | 0.5267  (1) | 0.4500  (1) | 0.6716  (1) | 0.6661  (1) | 4 | 13 |
| 10 | Intermediate | 2 | 0.4238  (0) | 0.0369  (0) | 1.1695  (0) | 0.3420  (1) | 1.2304  (0) | 0.2749  (0) | 1 | 3 |
| 11 | Sensitive | 0 | 0.3437  (0) | 0.0401  (0) | 1.0194  (0) | 0.3030  (1) | 0.7617  (0) | 0.4895  (0) | 1 | 1 |
| 12 | Sensitive | 1 | 0.7773  (0) | 0.0590  (0) | 0.8326  (0) | 0.1990  (0) | 0.9938  (0) | 0.3006  (0) | 0 | 1 |
| 13 | Sensitive | 3 | 0.1169  (1) | 0.0160  (1) | 0.2580  (1) | 0.4990  (1) | 0.3295  (1) | 0.7240  (1) | 6 | 9 |
| 14 | Sensitive | 5 | 0.2012  (0) | 0.0521  (0) | 0.4374  (1) | 0.2520  (0) | 0.4620  (1) | 0.5530  (1) | 3 | 8 |
| 15 | Resistant | 7 | 0.1714  (1) | 0.0267  (1) | 0.6582  (0) | 0.3110  (1) | 0.5489  (1) | 0.7534  (1) | 5 | 12 |
| 16 | Resistant | 7 | 0.1736  (1) | 0.0177  (1) | 0.3739  (1) | 0.4400  (1) | 0.4467  (1) | 0.5243  (1) | 6 | 13 |
| 17 | Intermediate | 3 | 0.2212  (0) | 0.0469  (0) | 0.6638  (0) | 0.1900  (0) | 0.4085  (1) | 0.3388  (0) | 1 | 4 |
| 18 | Sensitive | 6 | 0.2715  (0) | 0.0297  (0) | 0.4320  (1) | 0.2530  (0) | 0.8337  (0) | 0.2844  (0) | 1 | 7 |
| 19 | Intermediate | 0 | 0.4544  (0) | 0.0278  (0) | 0.6021  (0) | 0.0960  (0) | 0.8407  (0) | 0.2937  (0) | 0 | 0 |
| 20 | Intermediate | 6 | 0.1086  (1) | 0.0140  (1) | 0.3532  (1) | 0.3400  (1) | 0.4374  (1) | 0.4091  (0) | 5 | 11 |
