# Supplementary figures and images for: Distinctive serum lipidomic profile of IVIG-resistant Kawasaki disease children before and after treatment
Source: PLoS One. 2023 Mar 29;18(3):e0283710. doi: 10.1371/journal.pone.0283710 (PMC10057782; doi:10.1371/journal.pone.0283710)

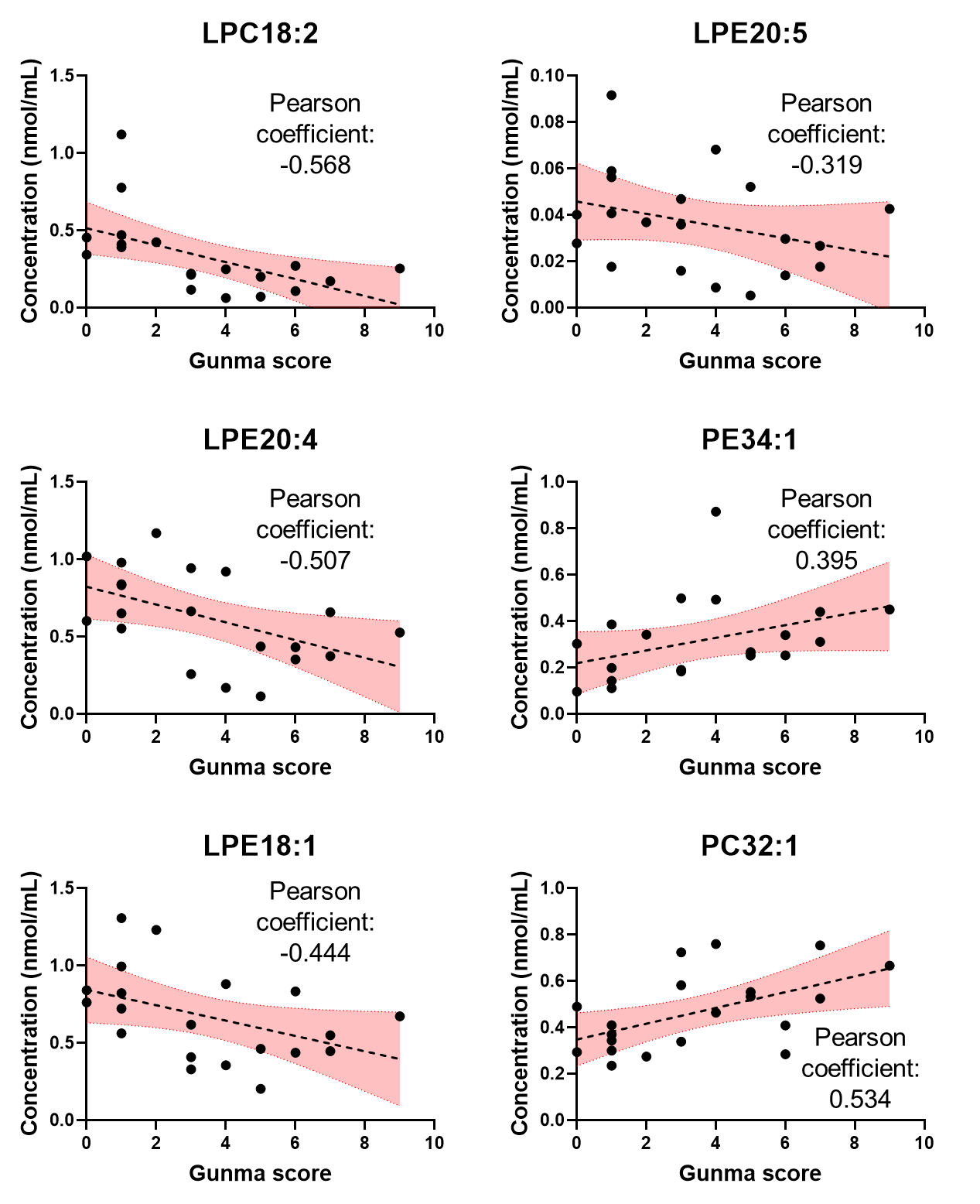


**S5 Fig.** Correlation between Gunma score and content of the potential lipid markers

Supplement: S1 Fig — (DOCX) [file pone.0283710.s006.docx]
